# Molecular phylogeny of the *Anopheles hyrcanus* group (Diptera: Culicidae) based on rDNA–ITS2 and mtDNA–COII

**DOI:** 10.1186/s13071-021-04971-4

**Published:** 2021-09-06

**Authors:** Canglin Zhang, Rui Yang, Linbo Wu, Chunhai Luo, Xiaofang Guo, Yan Deng, Hongning Zhou, Yilong Zhang

**Affiliations:** 1 Yunnan Provincial Key Laboratory of Vector-Borne Diseases Control and Research, Yunnan Provincial Collaborative Innovation Center for Public Health and Disease Prevention and Control, Yunnan Institute of Parasitic Diseases Innovative Team of Key Techniques for Vector Borne Disease Control and Prevention (Developing), Yunnan Institute of Parasitic Diseases, Pu’er, 665099 People’s Republic of China; 2 Department of Tropical Diseases, Faculty of Naval Medicine, Naval Medical University, Shanghai, 200433 People’s Republic of China

**Keywords:** *Anopheles*, Malaria, DNA barcoding, ITS2, COII, Phylogeny

## Abstract

**Background:**

The *Anopheles hyrcanus* group, which includes 25 species, is widely distributed in the Oriental and Palaearctic regions. Given the difficulty in identifying cryptic or sibling species based on their morphological characteristics, molecular identification is regarded as an important complementary approach to traditional morphological taxonomy. The aim of this study was to reconstruct the phylogeny of the Hyrcanus group using DNA barcoding markers in order to determine the phylogenetic correlations of closely related taxa and to compare these markers in terms of identification efficiency and genetic divergence among species.

**Methods:**

Based on data extracted from the GenBank database and data from the present study, we used 399 rDNA–ITS2 sequences of 19 species and 392 mtDNA–COII sequences of 14 species to reconstruct the molecular phylogeny of the Hyrcanus group across its worldwide range. We also compared the performance of rDNA–ITS2 against that of mtDNA–COII to assess the genetic divergence of closely related species within the Hyrcanus group.

**Results:**

Average interspecific divergence for the rDNA–ITS2 sequence (0.376) was 125-fold higher than the average intraspecies divergence (0.003), and average interspecific divergence for the mtDNA–COII sequence (0.055) was eightfold higher than the average intraspecies divergence (0.007). The barcoding gap ranged from 0.015 to 0.073 for rDNA–ITS2, and from 0.017 to 0.025 for mtDNA–COII. Two sets of closely related species, namely, *Anophels lesteri* and *An. paraliae*, and *An. sinensis*, *An. belenrae* and *An. kleini*, were resolved by rDNA–ITS2. In contrast, the relationship of *An. sinensis*/*An. belenrae*/*An. kleini* was poorly defined in the COII tree. The neutrality test and mismatch distribution revealed that *An. peditaeniatus*, *An. hyrcanus*, *An. sinensis* and *An. lesteri* were likely to undergo hitchhiking or population expansion in accordance with both markers. In addition, the population of an important *vivax* malaria vector, *An. sinensis*, has experienced an expansion after a bottleneck in northern and southern Laos.

**Conclusions:**

The topology of the Hyrcanus group rDNA–ITS2 and mtDNA–COII trees conformed to the morphology-based taxonomy for species classification rather than for that for subgroup division. rDNA–ITS2 is considered to be a more reliable diagnostic tool than mtDNA–COII in terms of investigating the phylogenetic correlation between closely related mosquito species in the Hyrcanus group. Moreover, the population expansion of an important *vivax* malaria vector,* An. sinensis*, has underlined a potential risk of malaria transmission in northern and southern Laos. This study contributes to the molecular identification of the *Anopheles hyrcanus* group in vector surveillance.

**Graphical abstract:**

**Figure:**
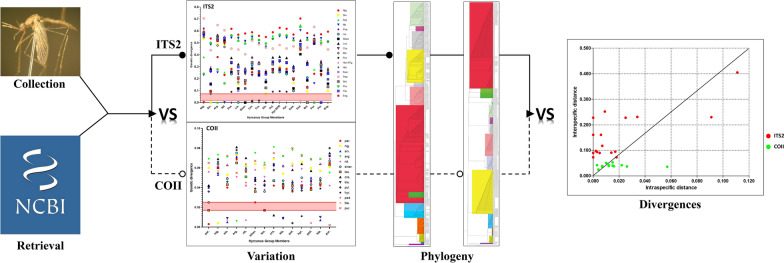

**Supplementary Information:**

The online version contains supplementary material available at 10.1186/s13071-021-04971-4.

## Background

The *Anopheles hyrcanus* group consists of at least 25 species and is classified into the Myzorhynchus series of *Anopheles*, with one provisional designated member [1, 2]. The members of this group are extensively distributed within the Oriental and Palaearctic regions, including a number of species capable of transmitting not only malaria [3–6] and filariasis [7, 8], but also Japanese encephalitis virus [9–11]. According to previous studies, *Anopheles sinensis* and *An. lesteri* are the major malaria vector present in China [12]; *An. hyrcanus* acts as a potential malaria vector in the south of France [13, 14]; *An. kleini* and *An. pullus* are the primary malaria vectors in South Korea [3]; *An. sinensis*, *An. nigerrimus* and *An. peditaeniatus* are the potential malaria vectors in Thailand [4]; *An. hyrcanus* group is among the major *Anopheles* species found across eight provinces in Laos [15]. As suggested in our recent study, *An. sinensis* is the predominant *Anopheles* species and suspected to be extensively distributed in the north of Laos (Phoshaly Province) [16]. Since the primary malaria vectors are classified mainly into the Hyrcanus group, it is essential to devise an efficient and precise method to identify the members of this group [17], which is an essential requirement for malaria vector surveillance in practice [18, 19]. However, even trained taxonomists are unlikely—or find it extremely difficult— to accurately distinguish species within the Hyrcanus group based only on morphological properties [6, 7] due to the significant variation in morphology and the adults of some closely related species exhibiting nearly identical adult morphological properties [8, 9].

DNA barcoding refers to an important addition to conventional approaches based on morphology and an effective tool that is used to identify species without the need to consider life stages. A DNA marker that is evolving at the species level can contribute toward accurate phylogenetic reconstruction in the Hyrcanus group and elucidate the ambiguity arising from an improper classification process [20, 21]. The internal transcribed spacer 2 (ITS2) has been commonly employed to address taxonomic problems in the Hyrcanus group due to its low intraspecific and high interspecific variability, as suggested in an abundance of studies [8, 9, 18, 22–24]. Using this marker, three newly proposed lineages revealed, including two species, *An. belenrae* and *An. kleini*, separated from *An. sinensis* [25], and one species showing a close relation to *An. hyrcanus*, with the provisional designation of *An. hyrcanus* sp_IR_ [18]. The mitochondrial cytochrome* c* oxidase subunit region (e.g. COI and COII) was taken as the standard barcode for identifying species within an extensive range of animal taxa [18, 19] and may be effective in providing barcoding data, in particular for assessing interspecific hybridization. Nevertheless, introgression in animals is considered to frequently involve mitochondrial DNA (mtDNA), as evidenced by recently appearing hybridization events among species [26, 27].

In order to reconstruct the molecular phylogeny of the Hyrcanus group, it is necessary to identify the barcoding gap of ITS2 and COII. Accordingly, the specimens of identical species in different geography sites must be examined [19, 28] for calculating the intraspecific and interspecific variations of COII and ITS2 within the group. The database of COII and ITS2 sequences in GenBank enables reference sequences to be used for identifying Hyrcanus group species based on a comparatively extensive geographic distribution [29]. Therefore, in the present study, GenBank sequences and data from our original study in northern and southern Laos were used to reconstruct a phylogeny for the Hyrcanus group on the basis of ITS2 and COII, with the aim to determine the phylogenetic correlations between taxa with close relations. In addition, we compared rDNA–ITS2 and mtDNA–COII in terms of their efficiency to distinguish different species and to determine the genetic divergence among different species in the Hyrcanus group, thereby contributing to the identification of molecular data on mosquitoes for use in malaria vector surveillance.

## Methods

### Mosquito collection and identification

Adult mosquitoes were collected by overnight trapping with battery-operated CDC light traps (model 1012; John W. Hock Inc., Gainesville, FL, USA) in cattle/pig pens or human residences (rooms) from 20:00 h to 08:00 h in Pathoomphone County (Champasak Province), Pak lay County (Xaignabouli Province) and Yot Ou County (Phongsaly Province), Laos, in 2018 (Additional file 1: Fig. S1). The live adult mosquitoes were killed by freezing. Subsequent isolation and identification procedures were carried out based on gender, species and subgroup, under a dissecting microscope using standard techniques [30, 31]. All mosquitoes were first morphologically sorted in the field using the keys of Das et al. [32]. Each morphologically identified specimen was kept individually in a 1.5-ml microcentrifuge tube filled with 75% ethanol and then stored at 4 ℃ for molecular confirmation of species and further processing.

### DNA extraction, ITS2/COII amplification and sequencing

Genomic DNA was isolated from individual mosquitoes using the QIAamp® DNA Mini Kit (QIAGEN, Hilden, Germany) following the manufacturer’s instructions. Approximately 650 bp of the COII gene and a 550-bp PCR product of the ITS2 region was amplified using primer pairs LYS-R (5′-ACTTGCTTTCAGTCATCTAATG-3′) and LEU-F (5′-TCTAATATGGCAGATTAGTGCA-3′) and ITS2-R (5′-TATGCTTAAATTCAGGGGGT-3′) and ITS2-F (5′-TGTGAACTGCAGGACACAT-3′), respectively. ITS2 was amplified in a PCR reaction volume of 25 µl with the following cycling parameters: 94 ℃, 2 min; then 94 ℃/30 s, 50 ℃/30 s, 72 ℃/40 s for 40 cycles; and a final extension at 72 ℃ for 10 min. COII was amplified in a PCR reaction volume of 25 µl with the following cycling parameters: 95 ℃, 5 min; then 95 ℃/1 min, 51 ℃/1 min, 72 ℃/ 2 min for 35 cycles; wiath a final extension 72 ℃ for 10 min. The PCR products were then analyzed by 1.5% agarose gel electrophoresis stained with GoldView (Beijing Solarbio Science & Technology Co., Ltd., Beijing, China), under UV transillumination. The sequencing reaction proceeded in both directions with the assistance of an ABI Big Dye Terminator Kit v.3.1 (Applied Biosystems, Thermo Fisher Scientific, Waltham, MA, USA); analysis was conducted using ABI Prism 3500xL-Avant Genetic Analyzer (Applied Biosystems, Thermo Fisher Scientific) in Shanghai (Sangon Biotech).

### Sequence alignment and phylogenetic analysis

The keywords “(species name)” and “ITS2/COII” were used to search for ITS2 or COII sequences of members of the Hyrcanus group deposited in GenBank. ITS2 and COII sequences that were distant from conspecific sequences after initial sequence alignment were eventually excluded from further analyses. In total, 691 ITS2 sequences and 368 COII sequences of the Hyrcanus group were ultimately extracted from GenBank and used in this study (Additional file 2: Tables S1, S2). These sequences were subsequently aligned and identical sequences obtained from the same dataset or species were excluded from further analysis. Ultimately, 267 ITS2 and 260 COII sequences (haplotypes) were further screened in a genetic divergence analysis and phylogenetic analysis (Additional file 2: Tables S3, S4). A total of 132 COII and ITS2 sequences from 89 *An. sinensis*, 18 *An. peditaeniatus*, 3 *An. nitidus*, 8 *An. argyropus* and 14 *An. nigerrimus* were generated in this study. Table 1 provides detailed information on the ITSE and COII sequences.

**Table 1:** The ITS2 and COII sequences of the *Anopheles hyrcanus* group samples collected from northern and southern Laos

ID^a^	Species	Location	Latitude	Longitude
LCB9(Nig)	*Anopheles nigerrimus*	Champasak Province: Pathoomphone County	106°04′	14°43′
LCB30(Nig)	*Anopheles nigerrimus*	Champasak Province: Pathoomphone County	106°04′	14°43′
LCB38(Nig)	*Anopheles nigerrimus*	Champasak Province: Pathoomphone County	106°04′	14°43′
LCB42(Nig)	*Anopheles nigerrimus*	Champasak Province: Pathoomphone County	106°04′	14°43′
LCB52(Nig)	*Anopheles nigerrimus*	Champasak Province: Pathoomphone County	106°04′	14°43′
LCB56(Nig)	*Anopheles nigerrimus*	Champasak Province: Pathoomphone County	106°04′	14°43′
LCB72(Nig)	*Anopheles nigerrimus*	Champasak Province: Pathoomphone County	106°04′	14°43′
LCB75(Nig)	*Anopheles nigerrimus*	Champasak Province: Pathoomphone County	106°04′	14°43′
LCB76(Nig)	*Anopheles nigerrimus*	Champasak Province: Pathoomphone County	106°04′	14°43′
LCB79(Nig)	*Anopheles nigerrimus*	Champasak Province: Pathoomphone County	106°04′	14°43′
LCB87(Nig)	*Anopheles nigerrimus*	Champasak Province: Pathoomphone County	106°04′	14°43′
LCB90(Nig)	*Anopheles nigerrimus*	Champasak Province: Pathoomphone County	106°04′	14°43′
LCB93(Nig)	*Anopheles nigerrimus*	Champasak Province: Pathoomphone County	106°04′	14°43′
LCB100(Nig)	*Anopheles nigerrimus*	Champasak Province: Pathoomphone County	106°04′	14°43′
LPB1(Sin)	*Anopheles sinensis*	Xaignabouli Province: Pak lay County	101°82′	19°39′
LPB4(Sin)	*Anopheles sinensis*	Xaignabouli Province: Pak lay County	101°82′	19°39′
LCB25(Sin)	*Anopheles sinensis*	Champasak Province: Pathoomphone County	106°04′	14°43′
LCB47(Sin)	*Anopheles sinensis*	Champasak Province: Pathoomphone County	106°04′	14°43′
LCB55(Sin)	*Anopheles sinensis*	Champasak Province: Pathoomphone County	106°04′	14°43′
LCB73(Sin)	*Anopheles sinensis*	Champasak Province: Pathoomphone County	106°04′	14°43′
LPY2(Sin)	*Anopheles sinensis*	Phongsaly Province: Yot Ou County	101°79′	22°12′
LPY4(Sin)	*Anopheles sinensis*	Phongsaly Province: Yot Ou County	101°79′	22°12′
LPY8(Sin)	*Anopheles sinensis*	Phongsaly Province: Yot Ou County	101°79′	22°12′
LPY9(Sin)	*Anopheles sinensis*	Phongsaly Province: Yot Ou County	101°79′	22°12′
LPY10(Sin)	*Anopheles sinensis*	Phongsaly Province: Yot Ou County	101°79′	22°12′
LPY11(Sin)	*Anopheles sinensis*	Phongsaly Province: Yot Ou County	101°79′	22°12′
LPY13(Sin)	*Anopheles sinensis*	Phongsaly Province: Yot Ou County	101°79′	22°12′
LPY14(Sin)	*Anopheles sinensis*	Phongsaly Province: Yot Ou County	101°79′	22°12′
LPY15(Sin)	*Anopheles sinensis*	Phongsaly Province: Yot Ou County	101°79′	22°12′
LPY16(Sin)	*Anopheles sinensis*	Phongsaly Province: Yot Ou County	101°79′	22°12′
LPY17(Sin)	*Anopheles sinensis*	Phongsaly Province: Yot Ou County	101°79′	22°12′
LPY19(Sin)	*Anopheles sinensis*	Phongsaly Province: Yot Ou County	101°79′	22°12′
LPY21(Sin)	*Anopheles sinensis*	Phongsaly Province: Yot Ou County	101°79′	22°12′
LPY22(Sin)	*Anopheles sinensis*	Phongsaly Province: Yot Ou County	101°79′	22°12′
LPY23(Sin)	*Anopheles sinensis*	Phongsaly Province: Yot Ou County	101°79′	22°12′
LPY25(Sin)	*Anopheles sinensis*	Phongsaly Province: Yot Ou County	101°79′	22°12′
LPY26(Sin)	*Anopheles sinensis*	Phongsaly Province: Yot Ou County	101°79′	22°12′
LPY29(Sin)	*Anopheles sinensis*	Phongsaly Province: Yot Ou County	101°79′	22°12′
LPY30(Sin)	*Anopheles sinensis*	Phongsaly Province: Yot Ou County	101°79′	22°12′
LPY31(Sin)	*Anopheles sinensis*	Phongsaly Province: Yot Ou County	101°79′	22°12′
LPY33(Sin)	*Anopheles sinensis*	Phongsaly Province: Yot Ou County	101°79′	22°12′
LPY35(Sin)	*Anopheles sinensis*	Phongsaly Province: Yot Ou County	101°79′	22°12′
LPY36(Sin)	*Anopheles sinensis*	Phongsaly Province: Yot Ou County	101°79′	22°12′
LPY37(Sin)	*Anopheles sinensis*	Phongsaly Province: Yot Ou County	101°79′	22°12′
LPY38(Sin)	*Anopheles sinensis*	Phongsaly Province: Yot Ou County	101°79′	22°12′
LPY39(Sin)	*Anopheles sinensis*	Phongsaly Province: Yot Ou County	101°79′	22°12′
LPY41(Sin)	*Anopheles sinensis*	Phongsaly Province: Yot Ou County	101°79′	22°12′
LPY43(Sin)	*Anopheles sinensis*	Phongsaly Province: Yot Ou County	101°79′	22°12′
LPY44(Sin)	*Anopheles sinensis*	Phongsaly Province: Yot Ou County	101°79′	22°12′
LPY45(Sin)	*Anopheles sinensis*	Phongsaly Province: Yot Ou County	101°79′	22°12′
LPY46(Sin)	*Anopheles sinensis*	Phongsaly Province: Yot Ou County	101°79′	22°12′
LPY47(Sin)	*Anopheles sinensis*	Phongsaly Province: Yot Ou County	101°79′	22°12′
LPY48(Sin)	*Anopheles sinensis*	Phongsaly Province: Yot Ou County	101°79′	22°12′
LPY49(Sin)	*Anopheles sinensis*	Phongsaly Province: Yot Ou County	101°79′	22°12′
LPY50(Sin)	*Anopheles sinensis*	Phongsaly Province: Yot Ou County	101°79′	22°12′
LPY51(Sin)	*Anopheles sinensis*	Phongsaly Province: Yot Ou County	101°79′	22°12′
LPY52(Sin)	*Anopheles sinensis*	Phongsaly Province: Yot Ou County	101°79′	22°12′
LPY53(Sin)	*Anopheles sinensis*	Phongsaly Province: Yot Ou County	101°79′	22°12′
LPY54(Sin)	*Anopheles sinensis*	Phongsaly Province: Yot Ou County	101°79′	22°12′
LPY56(Sin)	*Anopheles sinensis*	Phongsaly Province: Yot Ou County	101°79′	22°12′
LPY57(Sin)	*Anopheles sinensis*	Phongsaly Province: Yot Ou County	101°79′	22°12′
LPY59(Sin)	*Anopheles sinensis*	Phongsaly Province: Yot Ou County	101°79′	22°12′
LPY60(Sin)	*Anopheles sinensis*	Phongsaly Province: Yot Ou County	101°79′	22°12′
LPY61(Sin)	*Anopheles sinensis*	Phongsaly Province: Yot Ou County	101°79′	22°12′
LPY62(Sin)	*Anopheles sinensis*	Phongsaly Province: Yot Ou County	101°79′	22°12′
LPY63(Sin)	*Anopheles sinensis*	Phongsaly Province: Yot Ou County	101°79′	22°12′
LPY64(Sin)	*Anopheles sinensis*	Phongsaly Province: Yot Ou County	101°79′	22°12′
LPY65(Sin)	*Anopheles sinensis*	Phongsaly Province: Yot Ou County	101°79′	22°12′
LPY66(Sin)	*Anopheles sinensis*	Phongsaly Province: Yot Ou County	101°79′	22°12′
LPY67(Sin)	*Anopheles sinensis*	Phongsaly Province: Yot Ou County	101°79′	22°12′
LPY69(Sin)	*Anopheles sinensis*	Phongsaly Province: Yot Ou County	101°79′	22°12′
LPY70(Sin)	*Anopheles sinensis*	Phongsaly Province: Yot Ou County	101°79′	22°12′
LPY79(Sin)	*Anopheles sinensis*	Phongsaly Province: Yot Ou County	101°79′	22°12′
LPY80(Sin)	*Anopheles sinensis*	Phongsaly Province: Yot Ou County	101°79′	22°12′
LPY81(Sin)	*Anopheles sinensis*	Phongsaly Province: Yot Ou County	101°79′	22°12′
LPY82(Sin)	*Anopheles sinensis*	Phongsaly Province: Yot Ou County	101°79′	22°12′
LPY83(Sin)	*Anopheles sinensis*	Phongsaly Province: Yot Ou County	101°79′	22°12′
LPY84(Sin)	*Anopheles sinensis*	Phongsaly Province: Yot Ou County	101°79′	22°12′
LPY85(Sin)	*Anopheles sinensis*	Phongsaly Province: Yot Ou County	101°79′	22°12′
LPY86(Sin)	*Anopheles sinensis*	Phongsaly Province: Yot Ou County	101°79′	22°12′
LPY87(Sin)	*Anopheles sinensis*	Phongsaly Province: Yot Ou County	101°79′	22°12′
LPY89(Sin)	*Anopheles sinensis*	Phongsaly Province: Yot Ou County	101°79′	22°12′
LPY90(Sin)	*Anopheles sinensis*	Phongsaly Province: Yot Ou County	101°79′	22°12′
LPY91(Sin)	*Anopheles sinensis*	Phongsaly Province: Yot Ou County	101°79′	22°12′
LPY93(Sin)	*Anopheles sinensis*	Phongsaly Province: Yot Ou County	101°79′	22°12′
LPY94(Sin)	*Anopheles sinensis*	Phongsaly Province: Yot Ou County	101°79′	22°12′
LPY95(Sin)	*Anopheles sinensis*	Phongsaly Province: Yot Ou County	101°79′	22°12′
LPY96(Sin)	*Anopheles sinensis*	Phongsaly Province: Yot Ou County	101°79′	22°12′
LPY97(Sin)	*Anopheles sinensis*	Phongsaly Province: Yot Ou County	101°79′	22°12′
LPY109(Sin)	*Anopheles sinensis*	Phongsaly Province: Yot Ou County	101°79′	22°12′
LPY121(Sin)	*Anopheles sinensis*	Phongsaly Province: Yot Ou County	101°79′	22°12′
LPY122(Sin)	*Anopheles sinensis*	Phongsaly Province: Yot Ou County	101°79′	22°12′
LPY123(Sin)	*Anopheles sinensis*	Phongsaly Province: Yot Ou County	101°79′	22°12′
LPY125(Sin)	*Anopheles sinensis*	Phongsaly Province: Yot Ou County	101°79′	22°12′
LPY126(Sin)	*Anopheles sinensis*	Phongsaly Province: Yot Ou County	101°79′	22°12′
LPY127(Sin)	*Anopheles sinensis*	Phongsaly Province: Yot Ou County	101°79′	22°12′
LPY129(Sin)	*Anopheles sinensis*	Phongsaly Province: Yot Ou County	101°79′	22°12′
LPY130(Sin)	*Anopheles sinensis*	Phongsaly Province: Yot Ou County	101°79′	22°12′
LPY131(Sin)	*Anopheles sinensis*	Phongsaly Province: Yot Ou County	101°79′	22°12′
LPY132(Sin)	*Anopheles sinensis*	Phongsaly Province: Yot Ou County	101°79′	22°12′
LPY133(Sin)	*Anopheles sinensis*	Phongsaly Province: Yot Ou County	101°79′	22°12′
LPY134(Sin)	*Anopheles sinensis*	Phongsaly Province: Yot Ou County	101°79′	22°12′
LPY6(Sin)	*Anopheles sinensis*	Phongsaly Province: Yot Ou County	101°79′	22°12′
LCB5(Arg)	*Anopheles argyropus*	Champasak Province: Pathoomphone County	106°04′	14°43′
LCB13(Arg)	*Anopheles argyropus*	Champasak Province: Pathoomphone County	106°04′	14°43′
LCB22(Arg)	*Anopheles argyropus*	Champasak Province: Pathoomphone County	106°04′	14°43′
LCB51(Arg)	*Anopheles argyropus*	Champasak Province: Pathoomphone County	106°04′	14°43′
LCB68(Arg)	*Anopheles argyropus*	Champasak Province: Pathoomphone County	106°04′	14°43′
LCB24(Arg)	*Anopheles argyropus*	Champasak Province: Pathoomphone County	106°04′	14°43′
LCB29(Arg)	*Anopheles argyropus*	Champasak Province: Pathoomphone County	106°04′	14°43′
LCB31(Arg)	*Anopheles argyropus*	Champasak Province: Pathoomphone County	106°04′	14°43′
LCB4(Nit)	*Anopheles nitidus*	Champasak Province: Pathoomphone County	106°04′	14°43′
LCB96(Nit)	*Anopheles nitidus*	Champasak Province: Pathoomphone County	106°04′	14°43′
LCB99(Nit)	*Anopheles nitidus*	Champasak Province: Pathoomphone County	106°04′	14°43′
LCB6(Ped)	*Anopheles peditaeniatus*	Champasak Province: Pathoomphone County	106°04′	14°43′
LCB8(Ped)	*Anopheles peditaeniatus*	Champasak Province: Pathoomphone County	106°04′	14°43′
LCB17(Ped)	*Anopheles peditaeniatus*	Champasak Province: Pathoomphone County	106°04′	14°43′
LCB20(Ped)	*Anopheles peditaeniatus*	Champasak Province: Pathoomphone County	106°04′	14°43′
LCB21(Ped)	*Anopheles peditaeniatus*	Champasak Province: Pathoomphone County	106°04′	14°43′
LCB32(Ped)	*Anopheles peditaeniatus*	Champasak Province: Pathoomphone County	106°04′	14°43′
LCB40(Ped)	*Anopheles peditaeniatus*	Champasak Province: Pathoomphone County	106°04′	14°43′
LCB43(Ped)	*Anopheles peditaeniatus*	Champasak Province: Pathoomphone County	106°04′	14°43′
LCB45(Ped)	*Anopheles peditaeniatus*	Champasak Province: Pathoomphone County	106°04′	14°43′
LCB48(Ped)	*Anopheles peditaeniatus*	Champasak Province: Pathoomphone County	106°04′	14°43′
LCB50(Ped)	*Anopheles peditaeniatus*	Champasak Province: Pathoomphone County	106°04′	14°43′
LCB57(Ped)	*Anopheles peditaeniatus*	Champasak Province: Pathoomphone County	106°04′	14°43′
LCB62(Ped)	*Anopheles peditaeniatus*	Champasak Province: Pathoomphone County	106°04′	14°43′
LCB64(Ped)	*Anopheles peditaeniatus*	Champasak Province: Pathoomphone County	106°04′	14°43′
LCB65(Ped)	*Anopheles peditaeniatus*	Champasak Province: Pathoomphone County	106°04′	14°43′
LCB69(Ped)	*Anopheles peditaeniatus*	Champasak Province: Pathoomphone County	106°04′	14°43′
LCB86(Ped)	*Anopheles peditaeniatus*	Champasak Province: Pathoomphone County	106°04′	14°43′
LCB95(Ped)	*Anopheles peditaeniatus*	Champasak Province: Pathoomphone County	106°04′	14°43′

^a^LCB, Pathoomphone County (Champasak Province); LPB, Boun Neua County (Xaignabouli Province); LPY, Yot Ou County (Phongsaly Province)l Nig, *Anopheles nigerrimus*; Sin, *Anopheles sinensis*; Arg*, Anopheles argyropus*; Nit, *Anopheles nitidus*; Ped, *Anopheles peditaeniatus*.

The ITS2 and COII sequence dataset was combined with data on fragments from our original study and records retrieved from GenBank. A multiple sequence alignment was conducted in MEGA-X [33], while the manual adjustment was made using BioEdit V7.0.9 if required [34]. Gaps were excluded from the analysis and characters were unweighted. Both the maximum likelihood (ML) tree and the Neighbor-Joining (NJ) tree were performed with 1000 bootstraps in MEGA X [33]. The NJ method generally reveals shallow intraspecific and deep interspecific divergences [19, 35], for which a bootstrapped NJ tree was constructed using 1000 replicates [36] to provide a graphical representation of the phylogenetic correlations among the Hyrcanus group members. *Anopheles lindesayi* (GenBank accession no. AJ620898) and *An. claviger* (GenBank accession nos. AY129232 and DQ229313) were taken as outgroup taxa to the Hyrcanus group, in line with a prior study [37]. The visualization and the editing of the tree were performed using FigTree v1.4.2 [38].

### Genetic diversity analysis, demographic history analysis and neutrality test

Intra- and interspecific ITS2/COII divergences were tested using the Kimura’s 2-parameter (K2P) distance model [39] in MEGA X [33]. Genetic divergence was determined using Nei’s distance model [40], in Arlequin v3.5.2.2 [41]. Genetic diversity indices were calculated and neutrality tests (Fu’s Fs [42] and Tajima’s* D* [43]) were performed using DnaSP v5.10 [44]. The mismatch distribution (with the simulation in Arlequin v.3.5) was achieved to distinguish a multimodal or ragged distribution from a smooth unimodal distribution [45–47].

## Results

### Intra- and interspecific ITS2/COII variation

**Fig. 1:**
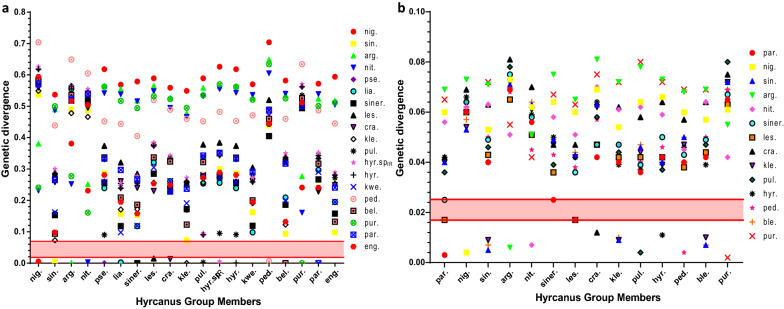
Intra- and interspecific divergences in the Hyrcanus group members determined using Kimura’s 2-parameter distance. Genetic divergence is shown on the* Y*-axis, and the Hyrcanus group members are shown on the* X*-axis. **a** Genetic divergence of ITS2. The barcoding gap ranged from 0.015 to 0.073. **b** Genetic divergence of COII. The barcoding gap ranged from 0.017 to 0.025. *arg.* *An. argyropus*, *bel.* *An. belenrae*, *cra.* *An. crawfordi*, *eng.* *An. engarensis*, *hyr.* *An. hyrcanus*, *hyr.* sp_IR_ *An. hyrcanus* sp_IR_, *kle.* *An. kleini*, *kwe.* *An. kweiyangensis*, *les.* *An. lesteri*, *lia*. *An. liangshanensis*, *nig.* *An. nigerrimus*, *nit.* *An. nitidus*, *par.* *An. paraliae*, *ped.* *An. peditaeniatus*, *pse.* *An. pseudopictus*, *pul.* *An. pullus*, *pur.* *An. pursati*, *siner.* *An. sineroides*, *sin.* *An. sinensis*

The mean intra- and interspecific K2P distances of the ITS2 sequence in 19 Hyrcanus group members and those of the COII sequence in 14 Hyrcanus group members were computed and compared using the K2P distance model [39] in this study. Tables 2 and 3 show the intra- and interspecific divergences of ITS2 and COII in the Hyrcanus group. Individual species were represented as few as one and as many as 143 individuals, for a total of 399 ITS2 sequences, and by one to 140 individuals for a total of 392 COII sequences. The distribution of the pairwise K2P genetic distance of ITS2 and COII is illustrated in Fig. 1, which shows a distinct barcoding gap.

**Table 2:** Mean intra- and interspecific K2P distances of the ITS2 sequence in 19 Hyrcanus group members

Species	*n*	*nig*	*sin*	*arg*	*nit*	*pse*	*lia*	*siner*	*les*	*cra*	*kle*	*pul*	*hyr.* sp_IR_	*hyr*	*kwe*	*ped*	*bel*	*pur*	*par*	*eng*
*nig*	24	**0.005**																		
*sin*	143	0.537	**0.004**																	
*arg*	12	0.381	0.490	**0.001**																
*nit*	8	0.231	0.487	0.252	**0.003**															
*pse*	2	0.618	0.286	0.565	0.542	**0.000**														
*lia*	3	0.569	0.157	0.555	0.538	0.239	**0.002**													
*siner*	7	0.579	0.153	0.518	0.516	0.255	0.118	**0.000**												
*les*	21	0.589	0.284	0.565	0.548	0.374	0.321	0.285	**0.015**											
*cra*	16	0.559	0.258	0.527	0.495	0.331	0.261	0.240	0.230	**0.013**										
*kle*	7	0.549	0.073	0.478	0.466	0.260	0.171	0.172	0.256	0.249	**0.000**									
*pul*	23	0.589	0.282	0.559	0.532	0.090	0.254	0.277	0.378	0.335	0.257	**0.002**								
*hyr.* sp_IR_	3	0.626	0.300	0.564	0.555	0.020^a^	0.256	0.277	0.384	0.343	0.272	0.095	**0.005**							
*hyr*	32	0.618	0.286	0.564	0.543	0.001^a^	0.239	0.255	0.374	0.331	0.260	0.091	0.020^a^	**0.001**						
*kwe*	1	0.570	0.162	0.528	0.536	0.259	0.098	0.119	0.305	0.262	0.191	0.281	0.273	0.259	**na**					
*ped*	73	0.704	0.439	0.649	0.605	0.452	0.444	0.405	0.520	0.489	0.460	0.452	0.482	0.453	0.471	**0.006**				
*bel*	8	0.582	0.094	0.536	0.540	0.337	0.209	0.186	0.337	0.324	0.123	0.322	0.350	0.336	0.200	0.460	**0.000**			
*pur*	4	0.241	0.500	0.277	0.161	0.562	0.517	0.494	0.532	0.523	0.495	0.535	0.570	0.562	0.521	0.634	0.525	**0.000**		
*par*	11	0.572	0.265	0.524	0.539	0.333	0.297	0.267	0.042^a^	0.228	0.237	0.343	0.352	0.333	0.291	0.487	0.317	0.501	**0.000**	
*eng*	1	0.594	0.098	0.517	0.505	0.281	0.195	0.158	0.254	0.249	0.069^a^	0.272	0.289	0.281	0.195	0.445	0.132	0.512	0.241	**na**

Intraspecific distances are shown in boldface for clarity. Underlined distances indicate the highest intraspecific distance and the lowest interspecific distance

*n*, no. of sequences; na, not applicable; *arg.*, *An. argyropus*; *bel.*, *An. belenrae*; *cra.*, *An. crawfordi*; *eng.*, *An. engarensis*; *hyr.* sp_IR_, *An. hyrcanus* sp_IR_; *hyr.*, *An. hyrcanus*; *kle.*, *An. kleini*; *kwe.*, *An. kweiyangensis*; *les.*, *An. lesteri*; *lia.*, *An. liangshanensis*; *nig.*, *An. nigerrimus*; *nit.*, *An. nitidus*; *par.*, *An. paraliae*; *ped.*, *An. peditaeniatus*; *pse.*, *An. pseudopictus*; *pul.*, *An. pullus*; *pur.*, *An. pursati*; *siner.*, *An. sineroides*; *sin.*, *An. sinensis*

^a^Interspecific distances of *hyrcanus*/*pseudopictus*, *hyrcanus*/*hyrcanus* sp_IR_, *hyrcanus* sp_IR_/*pseudopictus*, *lesteri*/*paraliae* and *kleini*/*engarensis*

**Table 3:** Mean intra- and interspecific K2P distances of the COII sequence in 14 Hyrcanus group members

Species	*n*	*par*	*nig*	*sin*	*arg*	*nit*	*siner*	*les*	*cra*	*kle*	*pul*	*hyr*	*ped*	*ble*	*pur*
*par*	8	**0.003**													
*nig*	24	0.060	**0.004**												
*sin*	140	0.040	0.053	**0.005**											
*arg*	13	0.069	0.073	0.071	**0.006**										
*nit*	21	0.056	0.062	0.063	0.051	**0.007**									
*siner*	1	0.025	0.064	0.049	0.075	0.058	**na**								
*les*	45	0.017^a^	0.060	0.043	0.065	0.051	0.036	**0.017**							
*cra*	29	0.042	0.069	0.063	0.081	0.070	0.047	0.047	**0.012**						
*kle*	16	0.040	0.054	0.009^a^	0.072	0.061	0.047	0.042	0.062	**0.009**					
*pul*	5	0.036	0.064	0.046	0.078	0.062	0.039	0.042	0.058	0.044	**0.004**				
*hyr*	30	0.042	0.066	0.040	0.073	0.059	0.050	0.042	0.064	0.039	0.037	**0.011**			
*ped*	54	0.040	0.060	0.050	0.068	0.045	0.043	0.038	0.057	0.047	0.040	0.046	**0.004**		
*ble*	3	0.042	0.057	0.007^a^	0.069	0.064	0.049	0.044	0.064	0.010^a^	0.047	0.039	0.050	**0.007**	
*pur*	3	0.065	0.061	0.072	0.055	0.042	0.067	0.063	0.075	0.072	0.080	0.072	0.069	0.069	**0.002**

Intraspecific distances are shown in boldface for clarity. Underlined distances indicate the highest intraspecific distance and the lowest interspecific distance

*n*, no. of sequences; na, not applicable; other abbreviations are as given in footnote to Table 2

^a^The interspecific distances of *lesteri*/*paraliae* and *sinensis*/*belenrae*/*kleini*

The intraspecific K2P distance of ITS2 reached 0.003 on average. However, no intraspecific variation was found for *An. belenrae*, *An. kleini*, *An. paraliae* or *An. pursati*. A high level of divergence was detected in two species: *An. lesteri* (0.015) and *An. crawfordi* (0.013). A short distance was detected in a number of species pairs, including *An. hyrcanus*/*An. pseudopictus* (0.001), *An. hyrcanus*/*An. hyrcanus* sp_IR_ (0.020), *An. hyrcanus* sp_IR_/*An. pseudopictus* (0.020), *An. lesteri*/*An. paraliae* (0.042) and *An. kleini*/*An. engarensis* (0.069) (Table 2). The taxonomic validity of *An. pseudopictus*, *An. hyrcanus*, *An. hyrcanus* sp_IR_, *An. paraliae* and *An. kleini* remains debatable [1, 9, 14, 18, 29], and further studies are required before a definitive conclusion can be drawn. Accordingly, the interspecific K2P distance varied from 0.073 between *An. kleini* and *An. sinensis* to 0.704 between *An. nigerrimus* and *An. pseudopictus*, with an average of 0.376. Based on these findings, the intragroup species divergence in the ITS2 sequence was approximately 125-fold higher than the average within-species divergence.

The intraspecific K2P distance of COII was 0.007 on average. A high level of divergence was detected in three species: *An. lesteri* (0.017), *An. crawfordi* (0.012) and *An. hyrcanus* (0.011). Nevertheless, *lesteri*/*paraliae* and *sinensis*/*belenrae*/*kleini* exhibited a poorly stated relationship, with a significantly low value of pairwise distance between *An. lesteri* and *An. paraliae* (0.017), *An. kleini* and *An. sinensis* (0.009), *An. belenrae* and *An. sinensis* (0.007) and *An. kleini* and *An. belenrae* (0.010) (Table 3). Accordingly, the interspecific K2P distance varied from 0.025 between *An. sineroides* and *An. paraliae* to 0.081 between *An. crawfordi* and *An. argyropus*, with an average of 0.055. Based on these findings, the intragroup species divergence in the COII sequence was approximately eightfold higher than the average within-species divergence.

**Fig. 2:**
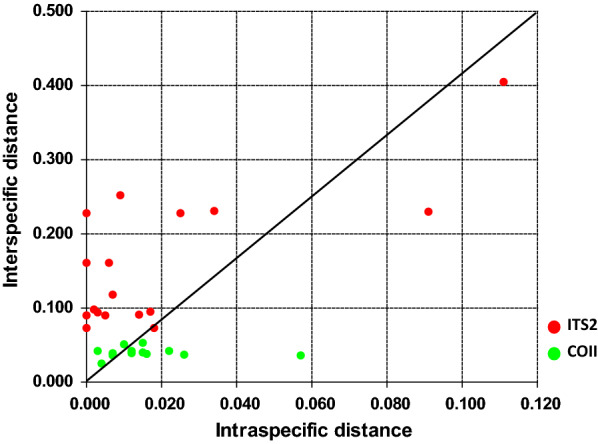
ITS2 and COII sequence divergences in the Hyrcanus group. The minimum interspecific (intergroup) divergence is plotted against the maximum intraspecific divergence. Red dots indicate the ITS2 sequence divergences of 16 species, including *An. argyropus*, *An. belenrae*, *An. crawfordi*, *An. hyrcanus*, *An. hyrcanus* sp_IR_, *An. kleini*, *An. lesteri*, *An. liangshanensis*, *An. nigerrimus*, *An. nitidus*, *An. paraliae*, *An. peditaeniatus*, *An. pullus*, *An. pursati*, *An. sineroides* and *An. sinensis*. Green dots indicate the COII sequence divergences of 13 species, including *An. argyropus*, *An. belenrae*, *An. crawfordi*, *An. hyrcanus*, *An. kleini*, *An. lesteri*, *An. nigerrimus*, *An. nitidus*, *An. paraliae*, *An. peditaeniatus*, *An. pullus*, *An. pursati* and *An. sinensis*

The ITS2 barcoding gap ranged from 0.015 to 0.073 (Fig. 1a), while the COII barcoding gap ranged from 0.017 to 0.025 (Fig. 1b), suggesting that the ITS2 spacer can serve as a more effective marker than COII for differentiating members of the Hyrcanus group. In Fig. 2, each dot represents a species, with interspecific distance on the* Y*-axis and intraspecific distance on the *X*-axis. Notably, there are more ITS2 dots than COII dots close to the top left-hand corner of the graph.

### Phylogenetic analysis

ITS2 and COII records obtained from GenBank were combined with sequences obtained in the original study and suspicious fragments (those distant from conspecific sequences after initial sequence alignment) were excluded, leaving 399 ITS2 sequences of 19 members within the Hyrcanus group, together with 392 COII sequences of 14 Hyrcanus group members to reconstruct a phylogenetic tree. The topology of the NJ tree and ML tree showed an approximate consistency in terms of main lineage, despite a slight difference in node confidence data between the two (Additional file 3: Fig. S2; Additional file 4: Fig. S3). Although the ITS2- or COII-based phylogenetic tree was consistent with conventional morphology taxonomy in terms of species recognition, its subgroup arrangement failed to comply with that achieved under morphology-based grouping.

The NJ–K2P analysis of the ITS2 sequences resulted in the identification of two major clusters in the Hyrcanus group: the Nigerrimus and Lesteri-Unassigned species subgroups, respectively. The Nigerrimus subgroup includes *An. nigerrimus*, *An. nitidus*, *An. argyropus* and *An. pursati*, and the Lesteri-Unassigned species subgroup includes *An. lesteri*,* An. paraliae*, *An. crawford*, *An. peditaeniatus* (the Lesteri-subgroup), *An. sinensis*, *An. engarensis*, *An. belenrae*, *An. kleini*, *An. liangshanensis*, *An. kweiyangensis*, *An. sineroides*, *An. hyrcanus*, *An. hyrcanus* sp_IR_, *An. pseudopictus* and *An. pullus* (the Unassigned species subgroup). Each of these species was arranged on a single branch and had the homolog to its closest taxon in the tree, demonstrating their potential role as the candidate species or recent divergence. However, one *An. kweiyangensis* (GenBank accession no. AF261150.2) was classified into the *An. liangshanensis* clade, one *An. engarensis* (GenBank accession no. AB159604.1) was classified into the *An. klein* clade and one *An. hyrcanus* sp_IR_ was classified into the *An. hyrcanus* clade (Additional file 3: Fig. S2a). All lineages covering individuals representing the same species were supported by high bootstrap data, with the exceptions of *An. pseudopictus* and *An. hyrcanus*, which exhibited barcode congruence with a significantly small interspecific distance (0.001) (Tables 2, 3). Moreover, slight genetic divergence was also observed between *An. lesteri* and *An. paraliae* (0.042), between *An. kleini* and *An. engarensis* (0.069), between *An. liangshanensis* and *An. kweiyangensis* (0.098), between *An. hyrcanus* and *An. hyrcanus* sp_IR_ (0.020) and between *An. hyrcanus* sp_IR_ and *An. pseudopictus* (0.020) (Tables 2, 3).

According to the NJ-K2P analysis conducted on COII sequences, the group fell into a minimum of three major clusters. The first cluster comprised only *An. nigerrimus*; the second cluster included *An. nitidus*, *An. pursati* and *An. argyropus*; the third cluster included *An. sinensis*, *An. belenrae*, *An. kleini*, *An. lesteri*, *An. paraliae*, *An. crawfordi*, *An. hyrcanus*, *An. peditaeniatus*, *An. sineroides* and *An. pullus*. Nearly all those node-linking sequences of individuals pertaining to the identical species showed high bootstrap value; however, the correlation of *An. sinensis/An. belenrae/An. kleini* remained unclear. Instead, they exhibited extremely small pairwise distance data (Tables 2, 3) which led to the formation of a distinct clade with high node confidence data (Additional file 3: Fig. S2b). It is worth noting that five *An. lesteri* individuals (GenBank accession nos. EU699070.1, EU699071.1, EU699072.1, EU699073.1 and EU699065.1) were found much closer to *An. nitidus* individuals (GenBank accession nos. AB777833.1 and AB777824.1) than to some of their conspecifics; these were classified as *An. nitidus* lineage in the phylogenetic tree. Among these, EU699070.1 and EU699072.1 showed a 100% sequence similarity to AB777833.1;e EU699071.1 was 99.85% similar to AB777833.1; and EU699073.1 and EU699065.1 showed a 99.27% sequence similarity to AB777824.1. Nevertheless, the interspecific distance was 0.051 between *An. lesteri* and *An. nitidus*, which is close to the average interspecific distance (0.055). Thus, it is practically possible for these sequences from *An. lesteri* individuals to be incorrect, these results can presumably be attributed to the misidentification of original specimens.

### Demographic history and neutrality test on the basis of ITS2 and COII sequences

A demographic history and neutrality test was further conducted using a total of 823 ITS2 and 500 COII sequences of the Hyrcanus group extracted from GenBank and our original data. Tables 4 and 5 list the sequence numbers according to the respective species, haplotype diversity, haplotype number, polymorphic site and Fu’s Fs and Tajima’s* D*. The presence of positive neutrality test data indicates either balancing selection or population size reduction. In contrast, as suggested by a negative neutrality test value, the group underwent an expansion after a bottleneck was reached, which could be attributable to purifying the selection process or a selective sweep under the context of genetic hitchhiking.

**Table 4:** Genetic diversity indices and neutrality tests (Fu’s *Fs* and Tajima’s *D*) of the ITS2 region in 17 Hyrcanus group members

Species	N	S	Pi	H	Hd	Fu's *Fs*	Tajima's *D*
*An. paraliae*	26	–	–	1	–	–	–
*An. pursati*	22	–	–	1	–	–	–
*An. belenrae*	26	1	0.000200	2	0.077	− 1.094	− 1.15559
*An. peditaeniatus*	117	58	0.004080	11	0.18	− 2.072	− 2.67451***
*An. crawfordi*	53	12	0.011000	4	0.491	9.501	2.50567
*An. hyrcanus*	115	11	0.001670	9	0.509	− 4.462**	− 1.78638**
*An. hyrcanus* sp_IR_	7	6	0.00597	3	0.524	1.934	0.45159
*An. pullus*	33	2	0.000900	3	0.225	− 0.357	− 0.41482
*An. kleini*	13	–	–	1	–	–	–
*An. lesteri*	24	54	0.018260	10	0.62	1.406	− 1.88497**
*An. sineroides*	7	4	0.002710	4	0.714	− 1.217	− 1.43414
*An. liangshanensis*	4	1	0.001120	2	0.5	0.172***	− 0.61237
*An. pseudopictus*	30	–	–	1	–	–	–
*An. nitidus*	46	4	0.002250	4	0.648	1.009	0.42695
*An. argyropus*	26	4	0.004320	4	0.618	2.283	2.52775
*An. nigerrimus*	41	19	0.003140	4	0.45	2.075	− 2.105**
*An. sinensis*	231	17	0.003240	23	0.623	− 16.474***	− 1.60196*

Species represented by < 3 specimens were excluded from the analyses.

*, **, ***Significance of Fu’s *Fs* and Tajima’s *D* values at *P < 0.05, **P < 0.02 and ***P < 0.001

N, number of sequences; S, number of polymorphic sites; Pi, nucleotide diversity; H, number of haplotypes; Hd, haplotype diversity

**Table 5:** Genetic diversity indices and neutrality tests (Fu’s Fs and Tajima’s *D*) of the COII gene in 13 Hyrcanus group members

Species	N	S	Pi	H	Hd	Fu's *Fs*	Tajima's *D*
*An. paraliae*	21	5	0.00127	4	0.533	− 0.286	− 1.1345
*An. pursati*	11	2	0.00149	2	0.509	2.343	1.50194
*An. belenrae*	3	6	0.00584	3	1	0.134***	–
*An. peditaeniatus*	83	21	0.00322	17	0.815	− 7.932*	− 1.64465*
*An. hyrcanus*	35	45	0.01102	24	0.963	− 9.744**	− 1.31777^#^
*An. pullus*	6	6	0.00351	5	0.933	− 1.728	− 0.49605
*An. kleini*	16	20	0.00765	15	0.992	− 9.839**	− 0.84867
*An. crawfordi*	48	32	0.01177	21	0.863	− 2.955	0.20133
*An. lesteri*	45	73	0.01529	38	0.986	− 24.744***	− 1.68623*
*An. nitidus*	24	19	0.00602	21	0.986	− 18.427***	− 0.9986
*An. argyropus*	17	12	0.00561	10	0.919	− 2.458	0.00026
*An. nigerrimus*	27	33	0.00449	16	0.906	− 8.892**	− 2.38098**
*An. sinensis*	163	40	0.00436	52	0.866	− 55.03***	− 1.97306**

Species represented by < 3 specimens were excluded from the analyses.

#, *, **, ***Significance of Fu’s *Fs* and Tajima’s *D* values at #*P* < 0.10, *P < 0.05, **P < 0.02 and ***P < 0.001

N, number of sequences; S, number of polymorphic sites; Pi, nucleotide diversity; H, number of haplotypes; Hd, haplotype diversity

The significant negative values of the neutrality test were identified within *An. lesteri* (Tajima’s *D* = − 1.88497, *P* < 0.02), *An. sinensis* (Tajima’s *D* = − 1.60196, *P* < 0.05; Fu’s Fs = − 16.474, *P* < 0.001), *An. nigerrimus* (Tajima’s *D* = − 2.105, *P* < 0.02), *An. hyrcanus* (Tajima’s *D* = − 1.78638, *P* < 0.02; Fu’s Fs = − 4.462, *P* < 0.02) and *An. peditaeniatus* (Tajima’s *D* = − 2.67451, *P* < 0.001) on the basis of ITS2 (Table 4), as were *An. lesteri* (Tajima’s *D* = − 1.68623, *P* < 0.05; Fu’s Fs = − 24.744, *P* < 0.001), *An. sinensis* (Tajima’s *D* = − 1.97306, *P* < 0.02; Fu’s Fs = − 55.03, *P* < 0.001), *An. nigerrimus* (Tajima’s *D* = − 2.38098, *P* < 0.02; Fu’s Fs = − 8.892, *P* < 0.02), *An. hyrcanus* (Fu’s Fs = − 9.744, *P* < 0.02), *An. nitidus* (Fu’s Fs = − 18.427, *P* < 0.001), *An. peditaeniatus* (Tajima’s *D* = − 1.64465, *P* < 0.05; Fu’s Fs = − 7.932, *P* < 0.05) and *An. kleini* (Fu’s Fs = − 9.839, *P* < 0.02) on the basis of COII (Table 5).

A smooth and unimodal mismatch distribution was detected in *An. lesteri*, *An. sinensis*, *An. hyrcanus* and *An. peditaeniatus* using both markers, which conforms to the expected mismatch distributions under the sudden expansion model. In addition, a smooth and unimodal mismatch distribution was also detected not only in the population of *An. liangshanensis* on the basis of ITS2, but also in the population of *An. nigerrimus* and *An. nitidus* on the basis of COII (Additional file 5: Fig. S4).

Furthermore, the *An. sinensis* samples collected from Laos in this study showed significant negative values in the neutrality test on the basis of both markers: ITS2 (Tajima’s *D* = − 1.52504, *P* < 0.05; Fu’s Fs = − 8.158, *P* < 0.02), COII (Tajima’s *D* = − 1.82131, *P* < 0.05; Fu’s Fs = − 17.607, *P* < 0.001) (data not shown). A smooth and unimodal mismatch distribution of ITS2 and COII was detected in the *An. sinensis* population from Laos. As mentioned above, this population experienced expansion after reaching a bottleneck (Additional file 6: Fig. S5)

## Discussion

### Subdivision of the Hyrcanus group

Based on morphological properties, the Hyrcanus group can be classified into three subgroups [48, 49]: the Nigerrimus subgroup, including *An. nigerrimus*, *An. nitidus*, *An. pursati* and *An. pseudosinensis*; the Lesteri subgroup, including *An. lesteri*, *An. paraliae*, *An. peditaeniatus*, *An. crawfordi* and *An. vietnamensis*; and species in an unassigned subgroup. Given the difficulty in identifying cryptic species by morphological characteristics alone, molecular methods have been used as powerful tools to complement traditional morphological taxonomy [10, 11, 17]. However, the barcoding of DNA for all members of the Hyrcanus group has scarcely been performed. Fang et al. were the first to have reconstructed the molecular phylogeny and analyzed the genetic divergence of the Hyrcanus group through two DNA barcoding markers, namely, ITS2 and COI [50, 51]. Based on the GenBank database and their original study data, they used 461 ITS2 sequences of 19 species and 466 COI sequences of 18 species to reconstruct the molecular phylogeny of the Hyrcanus group across its worldwide geographic range [50, 51].

Similarly, both rDNA and mtDNA were used in the present study to perform DNA barcoding. So far, there are 691 ITS2 of 19 species and 368 COII of 14 species of the Hyrcanus group deposited in GenBank (Additional file 2: Table S1). The NJ tree obtained in this study supported the monophyly of the Hyrcanus group, but the subgroup arrangement based on the rDNA and mtDNA DNA markers failed to comply with that indicated by morphological characteristics. Based on ITS2 sequences, there were two major subgroups that could be recognized: the first group includes *An. nigerrimus*, *An. nitidus*, *An. argyropus* and *An. pursati*, and the second group includes *An. sinensis*, *An. engarensis*, *An. belenrae*, *An. kleini*, *An. liangshanensis*, *An. kweiyangensis*, *An. sineroides*, *An. lesteri, An. paraliae*, *An. crawford*, *An. peditaeniatus*, *An. hyrcanus*, *An. pseudopictus* and *An. pullus*. This result is consistent with the study conducted by Fang based on ITS2 sequences and suggests that the morphology-based Lesteri subgroup is not monophyletic [51]. In addition, as indicated by the tree topologies in prior studies [8, 9, 18], *An. crawfordi* had the smallest distance to *An. peditaeniatus*; however, according to the results of the study of Fang [51] and the present study, *An. crawfordi* was at a distance from *An. peditaeniatus*, but approached *An. lesteri* and *An. paraliae* to form a single clade in the NJ tree based on ITS2. Moreover, *An. pseudopictus* individuals were embedded in the *An. hyrcanus* lineage in the ITS2 tree, implying the probability that *An. hyrcanus* and *An. pseudopictus* are the same species, consistent with the findings of prior studies [14, 51]. Similarly, *An. engarensis* and *An. kleini* were found to be of the identical species according to the study of Hwang [9] and the current study. However, the results allow for some speculation on the relationship between *An. kweiyangensis* and *An. liangshanensis*. In the ITS2 tree built in the study of Fang [51] and in the present study, the two species were classified into the same clade, but they were classified into two different clades in the COI tree [50].

Notably, three major clusters were found using the COII sequences in this study: *An. nigerrimus* was separated from the Nigerrimus group to form the first cluster; *An. nitidus*, *An. pursati* and *An. argyropus* were included in the second cluster; and all remaining species were included in the third cluster. Fang et al. reported that the pairwise distance of *An. paraliae* and *An. lesteri* reached 0.019, but that it was impossible to distinguish the two species in the phylogenetic tree based on COI [50]. In this study, however, the two species were easily distinguishable in the NJ tree based on COII, despite the pairwise distance of *An. lesteri* and *An. paraliae* reaching as low as 0.017. In their study, Taai et al. considered *An. paraliae* to be a synonym of *An. lesteri*, based on the results of crossing experiments performed between these two species using data on species distributions, morphological variants, cytology and comparative DNA sequence analyses [29]. Therefore, *An. paraliae* and *An. lesteri* should be treated as two closely related species in this study. In addition, the relationship between *An. sinensis*, *An. belenrae* and *An. kleini* remained inconclusive in the COII tree. Showing significantly low pairwise distance values (Tables 2, 3), these species contributed to the formation of a monoclade with a high achievement of node confidence (Additional file 3: Fig. S2), which suggests the potential occurrence of ancient hybridization in the aforementioned three species with close correlations.

Due to the failure in previous studies to cover an appropriate number of species, it is difficult to detect subgroups in the phylogenetic tree, as a result of which no consistent results were produced in the current and prior studies on the genetic relationships of Hyrcanus group members. The genes submitted to GenBank were not properly presented, with a number of error sequences possibly in the database [52, 53]. In this study, some error sequences related to the authors who submitted these sequences to GenBank were used to conduct the phylogenetic analysis, which led to misidentification (See Results section Phylogenetic analysis). These error sequences must be removed from future phylogenetic analyses.

### Phylogenetic reconstructions with the use of COII and ITS2

An effective DNA marker must exhibit low intraspecific distances and large interspecific distances [54]. In this study, we found that the COII barcoding gap varied from 0.017 to 0.025, whereas that of ITS2 ranged from 0.015 to 0.073. The ITS2 interspecific genetic distance was 125-fold higher than that of the average intraspecies difference; in comparison, the average COII divergence among group of species was merely eightfold higher than that within species.

Since mtDNA is characterized by maternal inheritance, any offspring or hybrid would have only the mtDNA of the maternal species. Accordingly, hybridization can lead to shared or highly consistent sequences in the mitochondrial genome. Therefore, the major downside of employing COII for phylogenetics research of the Hyrcanus group is that COII fails to distinguish between those species with a close relationship, i.e. *An. sinensis*, *An. belenrae* and *An. kleini*. As suggested by the divergent mtDNA of *An. belenrae* and *An. kleini*, the mitochondrial genomes of incipient sibling species were sympatrically replaced by those of *An. sinensis* extensively in many regions. In general, recent hybridization can play a role in transferring mtDNA from one species to another to cause mtDNA variation [26, 55, 56]. Thus, results on mtDNA are considered to be more useful when speculating about the possibility of ancient hybridization in mosquito molecular phylogeny. In contrast, rDNA has been suggested to be more reliable than mtDNA in addressing evolutionary problems with the recently diverged taxa or cryptic species of mosquitoes [51] and in building species boundaries when these can not be addressed by using mtDNA. Hence, the ITS2 marker has been widely used for species identification and phylogenetic reconstruction for the Hyrcanus group [8, 9, 18, 22, 23].

### Demographic history of *An. sinensis* population

According to the results of the neutrality tests and mismatch distribution, a number of members of the Hyrcanus group, namely *An. peditaeniatus*, *An. hyrcanus*, *An. sinensis* and *An. lesteri*, are suspected to have experienced hitchhiking or population expansion based on both markers. These four species show an extensive distribution, and some have the potential to transmit malaria. In addition, the *An. sinensis* samples collected from Laos in this study showed significant negative values in the neutrality tests, according to both markers. A smooth and unimodal mismatch distribution of ITS2 and COII was observed in the population of *An. sinensis* from Laos, suggesting that the expansion occurred after a bottleneck was reached. According to several recent studies, *An. sinensis* acted as an important vector of local malaria outbreak, which highlights a potential risk of malaria transmission in endemic areas [57, 58]. Therefore, the potential transmission of *vivax* malaria by *An. sinensis* in the northern and southern regions of Laos deserves more attention.

## Conclusions

The topology of the ITS2 and COII trees of the Hyrcanus group conformed to the morphology-based taxonomy for species classification rather than for subgroup division. Compared to COII within the Hyrcanus group, rDNA–ITS2 can be considered to be the more reliable tool for investigating the phylogenetic correlation of closely related mosquito species, such as *An. belenrae*, *An. kleini* and *An. sinensis*. Moreover, the population expansion of an important *vivax* malaria vector,* An. sinensis*, has been emphasized as a potential risk factor in malaria transmission in northern and southern Laos. The results of this study support the efficacy of molecular identification of the *Anopheles hyrcanus* group in terms of vector surveillance.

## Supplementary Information

**Additional file 1: Figure S1.** Map of the three sampling sites in Laos: Pathoomphone County (Champasak Province, Laos-Cambodia border), Pak lay County (Xaignabouli Province, Laos-Thailand border) and Yot Ou County (Phongsaly Province, Laos–China border). The shapefile map of Lao PDR was downloaded and prepared by using Pixelmap Generator-Beta on line (amCharts, Vilnius, Lithuania) (https://pixelmap.amcharts.com/), which is copyright free.

**Additional file 2: Table S1.** Full list of the 691 ITS2 sequences of the *Anopheles hyrcanus* group deposited in GenBank, with GenBank accession numbers, species, geographical location and corresponding authors. **Table S2.** Full list of 368 COII sequences of the *Anopheles hyrcanus* group deposited in GenBank, with GenBank accession numbers, species, geographical location and corresponding authors. **Table S3.** Full list of 267 ITS2 sequences (haplotypes) of the *Anopheles hyrcanus* group used for genetic divergence analysis and phylogenetic analysis. **Table S4.** Full list of 260 COII sequences (haplotypes) of the *Anopheles hyrcanus* group used for genetic divergence analysis and phylogenetic analysis.

**Additional file 3: Figure S2.** Neighbor-joining phylogenetic tree of the Hyrcanus group based on ITS2 (**a**) and COII sequences (**b**) from GenBank and our original data. Bootstrap values (1000 replicates) of neighbor-joining analyses are shown above/below the main lineages. Lineage designation is indicated on the right. Bars represent 20.00 substitutions per site based on ITS2 and 5.0 substitutions per site based on COII. *Anopheles lindesayi* and *An. claviger* were used as the outgroup taxa.

**Additional file 4: Figure S3.** Maximum Likelihood phylogenetic tree of the Hyrcanus group based on ITS2 (**a**) and COII sequences (**b**) from GenBank and our original data. Bootstrap values (1000 replicates) of maximum-likelihood analyses are shown above/below the main lineages. Lineage designation is indicated on the right. Bars represent 20.00 substitutions per site based on ITS2 and 7.0 substitutions per site based on COII. *Anopheles lindesayi* and *An. claviger* were used as the outgroup taxa.

**Additional file 5: Figure S4.** The mismatch distribution graphs in the Hyrcanus group based on ITS2 (**a**) and COII (**b**). The* X*- and* Y*-axis show the number of pairwise differences and the frequency of the pairwise comparisons, respectively. The observed frequencies are represented by a dotted line. The frequency expected under the hypothesis of constant population model is depicted by a solid line.*arg.* *An. argyropus*, *bel.* *An. belenrae*, *cra.* *An. crawfordi*, *eng.* *An. engarensis*, *hyr.* *An. hyrcanus*, *hyr.* sp_IR_ *An. hyrcanus* sp_IR_, *kle.* *An. kleini*, *kwe.* *An. kweiyangensis*, *les.* *An. lesteri*, *lia*. *An. liangshanensis*, *nig.* *An. nigerrimus*, *nit.* *An. nitidus*, *par.* *An. paraliae*, *ped.* *An. peditaeniatus*, *pse.* *An. pseudopictus*, *pul.* *An. pullus*, *pur.* *An. pursati*, *siner.* *An. sineroides*, *sin.* *An. sinensis*

**Additional file 6: Figure S5.** The mismatch distribution graphs in *Anopheles sinensis* based on ITS2 (**a**) and COII (**b**). The* X*- and* Y*-axis show the number of pairwise differences and the frequency of the pairwise comparisons, respectively. The observed frequencies are represented by a dotted line. The frequency expected under the hypothesis of constant population model is depicted by a solid line.

## Data Availability

Data supporting the conclusions of this article are included within the article and its additional files. The datasets generated and/or analysed during the current study are available in the GenBank (http://www.ncbi.nlm.nih.gov/). The raw datasets used and/or analysed during this study are available from the corresponding author upon reasonable request.

## Abbreviations

COII: Cytochrome* c* oxidase subunit 2
K2P: Kimura’s 2-parameter
ITS2: Internal transcribed spacer 2
mtDNA: Mitrochondrial DNA
ML: Maximum likelihood
NJ: Neighbor-joining
