# Supplementary figures and images for: Molecular phylogeny of the Anopheles hyrcanus group (Diptera: Culicidae) based on rDNA–ITS2 and mtDNA–COII
Source: Parasit Vectors. 2021 Sep 6;14:454. doi: 10.1186/s13071-021-04971-4 (PMC8420049; doi:10.1186/s13071-021-04971-4)

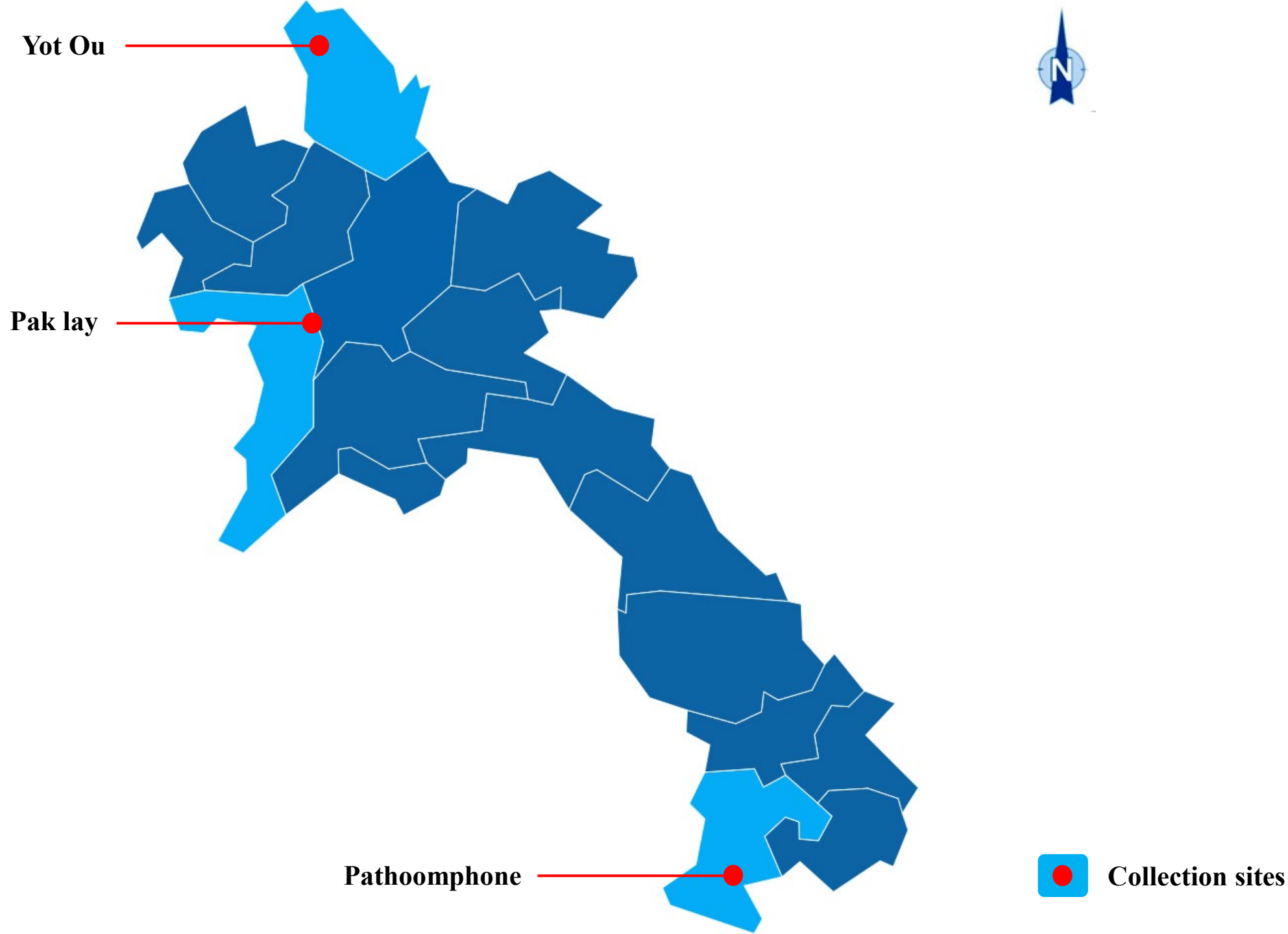

Supplement: Supplementary file 1 — Additional file 1: Figure S1. Map of the three sampling sites in Laos: Pathoomphone County (Champasak Province, Laos-Cambodia border), Pak lay County (Xaignabouli Province, Laos-Thailand border) and Yot Ou County (Phongsaly Province, Laos–China border). The shapefile map of Lao PDR was downloaded and prepared by using Pixelmap Generator-Beta on line (amCharts, Vilnius, Lithuania) (https://pixelmap.amcharts.com/), which is copyright free. [file 13071_2021_4971_MOESM1_ESM.pdf]

**a**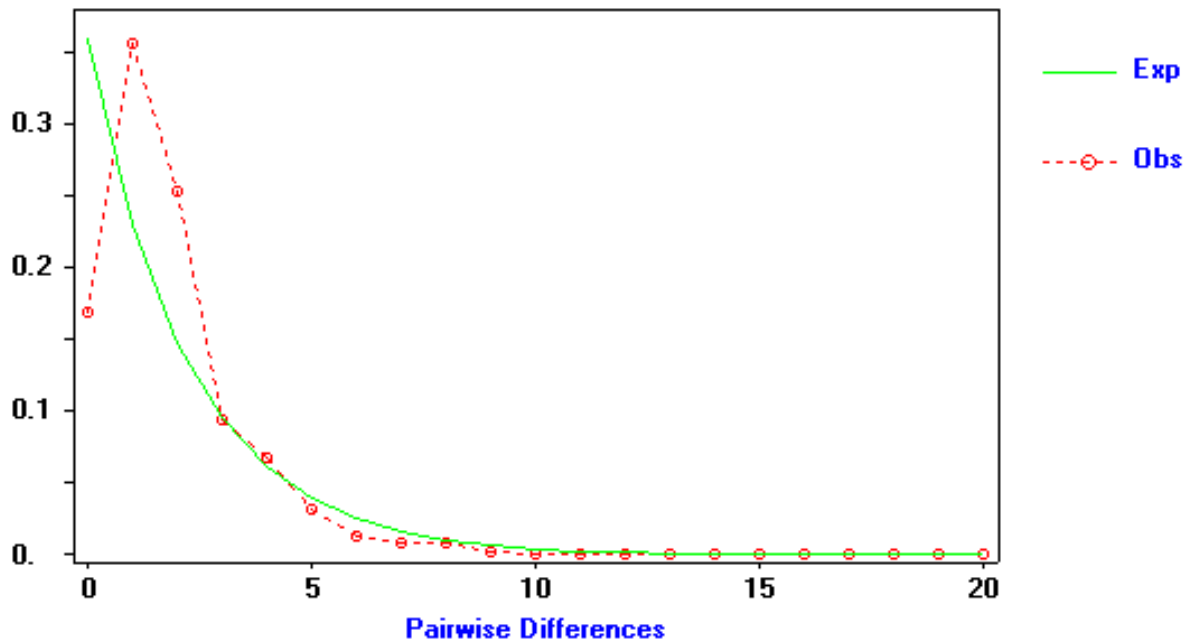**b**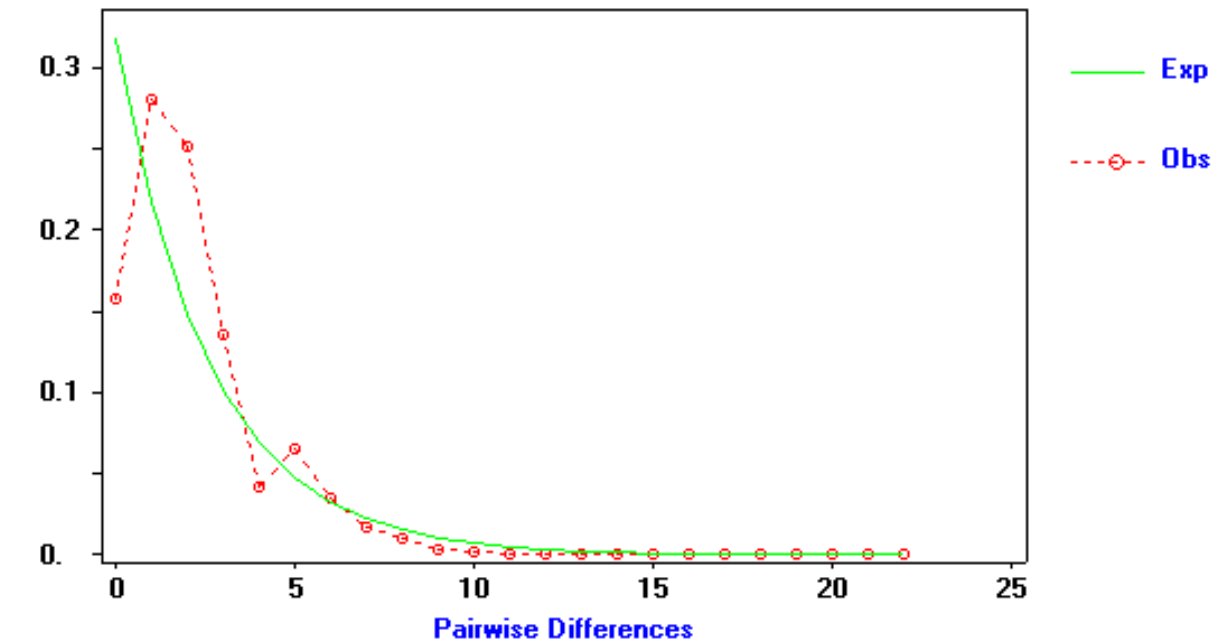

Supplement: Supplementary file 6 — Additional file 6: Figure S5. The mismatch distribution graphs in Anopheles sinensis based on ITS2 (a) and COII (b). The X- and Y-axis show the number of pairwise differences and the frequency of the pairwise comparisons, respectively. The observed frequencies are represented by a dotted line. The frequency expected under the hypothesis of constant population model is depicted by a solid line. [file 13071_2021_4971_MOESM6_ESM.pdf]
