# Supplementary material for: Molecular phylogeny of the Anopheles hyrcanus group (Diptera: Culicidae) based on rDNA–ITS2 and mtDNA–COII
Source: Parasit Vectors. 2021 Sep 6;14:454. doi: 10.1186/s13071-021-04971-4 (PMC8420049; doi:10.1186/s13071-021-04971-4)
Supplement: Supplementary file 5 — Additional file 5: Figure S4. The mismatch distribution graphs in the Hyrcanus group based on ITS2 (a) and COII (b). The X- and Y-axis show the number of pairwise differences and the frequency of the pairwise comparisons, respectively. The observed frequencies are represented by a dotted line. The frequency expected under the hypothesis of constant population model is depicted by a solid line.arg. An. argyropus, bel. An. belenrae, cra. An. crawfordi, eng. An. engarensis, hyr. An. hyrcanus, hyr. spIR An. hyrcanus spIR, kle. An. kleini, kwe. An. kweiyangensis, les. An. lesteri, lia. An. liangshanensis, nig. An. nigerrimus, nit. An. nitidus, par. An. paraliae, ped. An. peditaeniatus, pse. An. pseudopictus, pul. An. pullus, pur. An. pursati, siner. An. sineroides, sin. An. sinensis [file 13071_2021_4971_MOESM5_ESM.pdf]

a

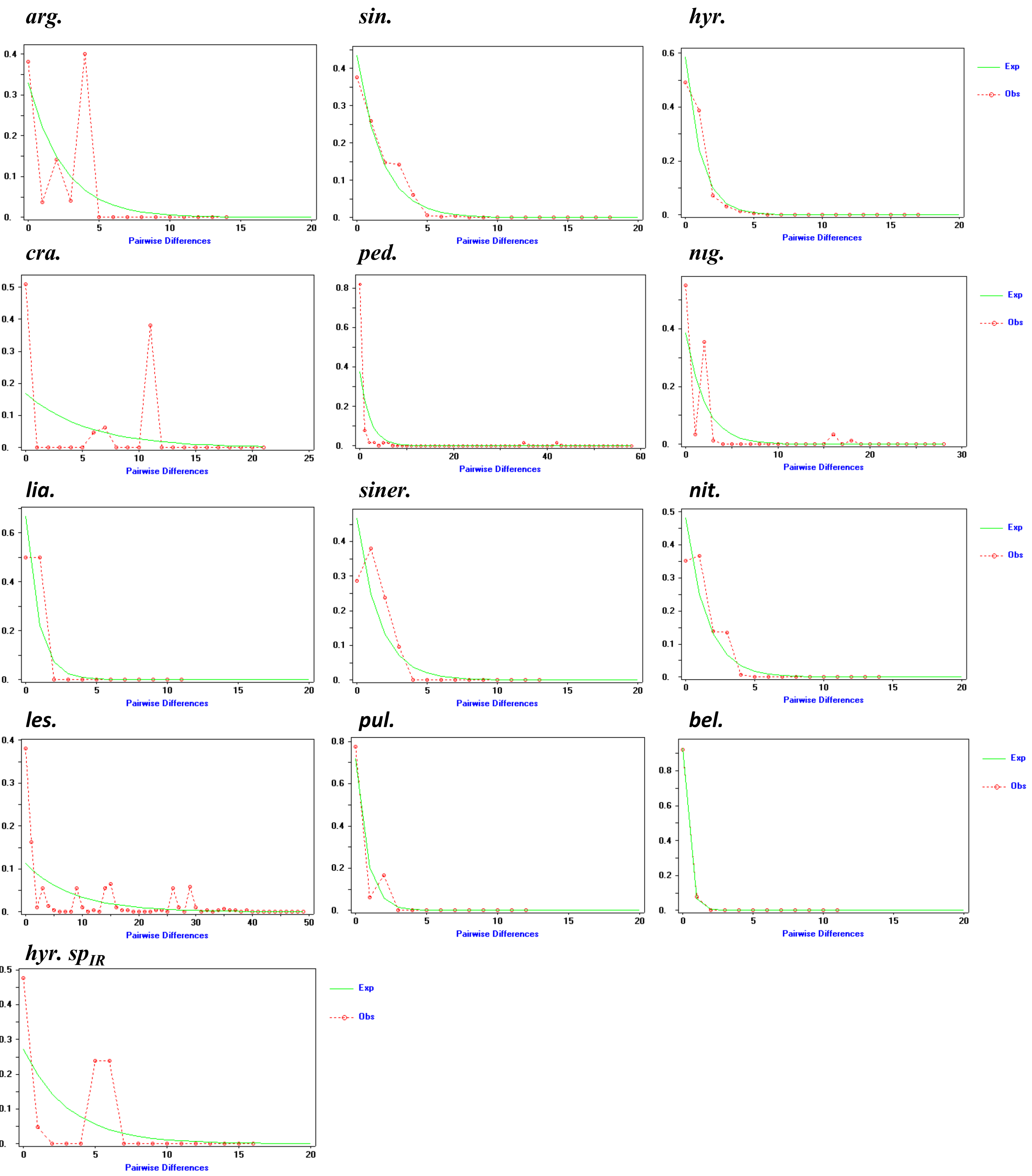
arg.
sin.
hyr.
cra.
ped.
nig.
lia.
siner.
nit.
les.
pul.
bel.
hyr. sp$_{IR}$
Pairwise Differences
Exp
Obs

b

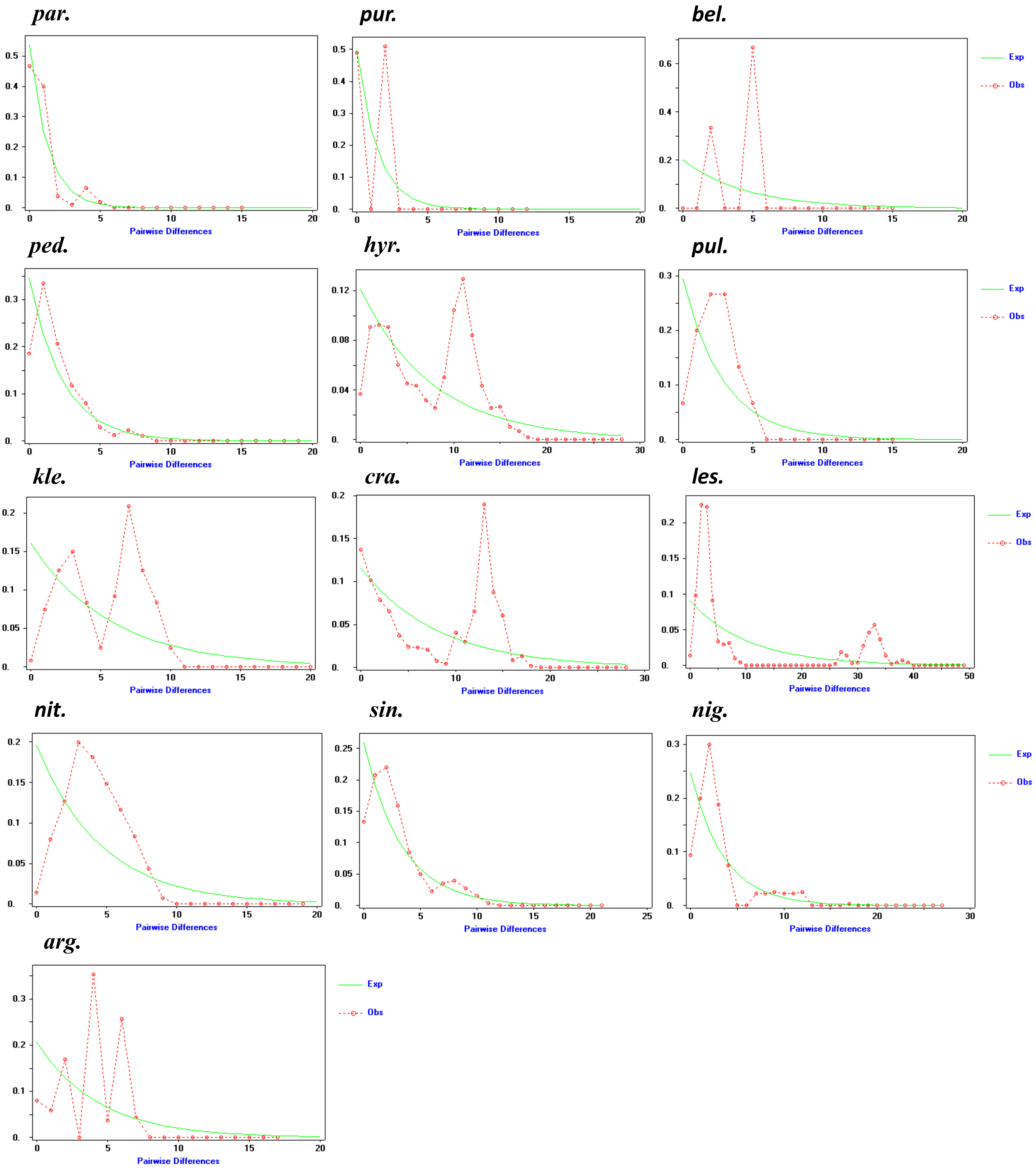

par.
pur.
bel.
ped.
hyr.
pul.
kle.
cra.
les.
nit.
sin.
nig.
arg.
Pairwise Differences
Exp
Obs
