# Supplementary material for: Molecular phylogeny of the Anopheles hyrcanus group (Diptera: Culicidae) based on rDNA–ITS2 and mtDNA–COII
Source: Parasit Vectors. 2021 Sep 6;14:454. doi: 10.1186/s13071-021-04971-4 (PMC8420049; doi:10.1186/s13071-021-04971-4)

a

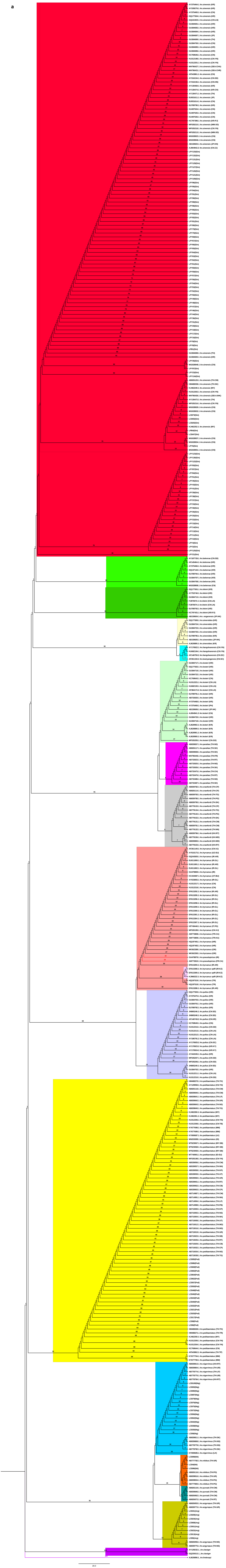

b

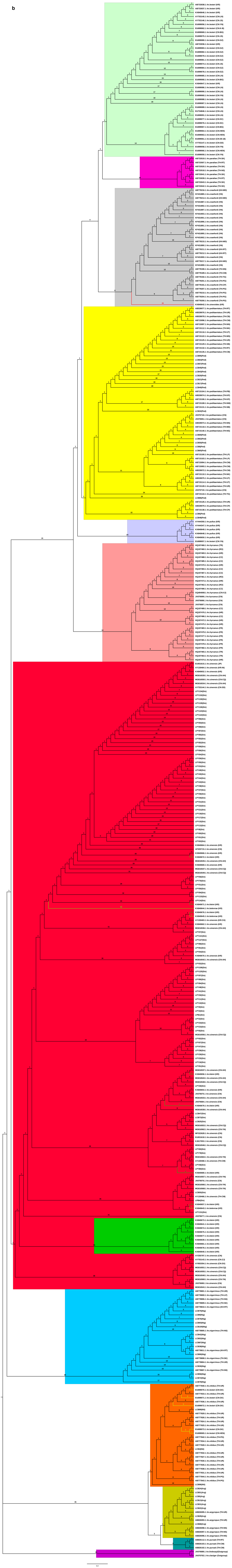

Supplement: Supplementary file 3 — Additional file 3: Figure S2. Neighbor-joining phylogenetic tree of the Hyrcanus group based on ITS2 (a) and COII sequences (b) from GenBank and our original data. Bootstrap values (1000 replicates) of neighbor-joining analyses are shown above/below the main lineages. Lineage designation is indicated on the right. Bars represent 20.00 substitutions per site based on ITS2 and 5.0 substitutions per site based on COII. Anopheles lindesayi and An. claviger were used as the outgroup taxa. [file 13071_2021_4971_MOESM3_ESM.pdf]
